# Enteral nutritional therapy practices in pediatric hematopoietic stem cell transplant centers in Brazil

**DOI:** 10.1016/j.htct.2026.108543

**Published:** 2026-10-16

**Authors:** Vitória Soares Viau, Andressa Florencio da Silva, Soraia Poloni, Vera Lucia Bosa, Estela Beatriz Behling

**Affiliations:** a Nutrition Program, School of Medicine, Federal University of Rio Grande do Sul, Porto Alegre, RS, Brazil; b Graduate Program in Food, Nutrition and Health, Federal University of Rio Grande do Sul, Porto Alegre, RS, Brazil; c Nutrition Service, Multiprofessional Residency Program in Health: Onco-Hematology, Hospital de Clínicas de Porto Alegre, Porto Alegre, RS, Brazil; d Nutrition Service, Multiprofessional Residency Program in Health: Women's and Children's Health, Hospital de Clínicas de Porto Alegre, Porto Alegre, RS, Brazil; e Department of Nutrition, School of Medicine, Federal University of Rio Grande do Sul, Porto Alegre, RS, Brazil

**Keywords:** Nutrition, Stem cell, transplantation, Enteral nutrition, Pediatric

## Abstract

**Introduction:**

The aim of this study was to analyze the use of enteral nutritional therapy in pediatric hematopoietic stem cell transplantation centers in Brazil, addressing its application, challenges, and current practices.

**Methods:**

This cross-sectional study was conducted from May to October 2023 in Brazilian pediatric hematopoietic stem cell transplantation centers. Data were collected via a structured questionnaire sent to eligible centers, covering the use of prophylactic nasoenteral tubes, formula selection, and adaptations for gastrointestinal symptoms.

**Results:**

Seventeen transplant centers participated, predominantly located in the Southeastern region (6.91%). Among them, 82.4% routinely used prophylactic nasoenteral tubes, with polymeric formulas being the first choice in 82.4% of cases. Normocaloric formulas (0.9–1.2 kcal/mL) were also widely adopted (82.4%). For managing gastrointestinal symptoms, the most common approach was reducing the diet volume (30.6%). This study identified a gap in specialized formulas for children aged 10 to 18 years, with 47.1% of centers opting for formulas designed for adults in this age group due to a lack of alternatives.

**Conclusions:**

The study highlights regional disparities in hematopoietic stem cell transplantation services in Brazil and confirms enteral nutritional therapy as the primary nutritional support in pediatric transplantations. It also reveals the absence of consensus regarding the best formulas and management protocols, particularly for the 10- to 18-year age group. The findings point to an urgent need for further research and the development of standardized guidelines to ensure safe and effective nutritional support, improving transplant outcomes, and promoting proper growth and development in these patients.

## Introduction

Hematopoietic Stem Cell Transplantation (HSCT), a therapeutic intervention for managing severe hematological disorders during childhood, including leukemias, severe anemias, and bone marrow failure syndromes, aims to restore bone marrow function through the infusion of progenitor cells. This procedure can be classified as either allogeneic or autologous HSCT, depending on the source of the progenitor cells [1,2]. In Brazil, 126 HSCT teams operate within 86 centers accredited by the Ministry of Health [3].

Patients undergoing myeloablative conditioning, particularly pediatric patients, are considered at nutritional risk and require early nutritional interventions. Effective nutritional therapy is essential to support growth demands, minimize deleterious treatment-related effects, and promote favorable clinical prognoses [1,2,[4], [5], [6]]

Although oral administration remains the preferred nutritional support route, gastrointestinal symptoms, hospital diet restrictions, and elevated metabolic demands often necessitate enteral nutritional therapy (ENT). ENT ensures adequate nutrient delivery, preserves intestinal mucosal integrity, and prevents complications like mucosal atrophy and bacterial translocation [2,4,7].

Rigorous monitoring allows adjustments and timely discontinuation of nutritional therapy to meet nutritional and growth needs. The early introduction of ENT reduces the need for parenteral nutrition, reserved for cases of gastrointestinal dysfunction with higher complication risks. ENT is associated with improved clinical outcomes and accelerated nutritional recovery [2,4,[6], [7], [8]].

The lack of standardized ENT protocols in pediatric HSCT leads to practices based mostly on individual experience. This study aims to map Brazilian centers and protocols, contributing to evidence-based guidelines to optimize ENT use and enhance recovery and prognosis in pediatric patients.

## Methods

This cross-sectional study analyzed Brazilian pediatric HSCT centers through an online questionnaire administered between May and October 2023. The study was approved by the Research Ethics Committee (CEP) of the Hospital de Clínicas de Porto Alegre (HCPA) under protocol number 56224422.0.0000.5327, and informed consent (TCLE) was obtained between June 12 and October 2, 2023.

The mapping of eligible centers was conducted using sources such as the website of the Brazilian Society for Cellular Therapy and Bone Marrow Transplantation (SBTMO), the Redome Network (INCA), and the websites of relevant associations including the Bone Marrow Association (AMEO), the Brazilian Association of Organ Transplantation (ABTO), and the Brazilian Association of Lymphoma and Leukemia (ABRALE). Following identification, centers were invited to participate via email, with follow-up telephone contact when necessary. Both public and private centers performing autologous and allogeneic pediatric transplants (ages 0–18) were eligible for inclusion. Centers conducting transplants exclusively in adults were excluded.

Among the 133 HSCT centers initially invited, 21 reported conducting pediatric HSCT; consequently, these were considered eligible for this study. Of these, 17 completed the questionnaire and were included in the final analysis (Figure 1). The questionnaire, distributed via Google Forms, consisted of four sections:

• Invitation, study presentation, and participant email collection;
• Informed Consent Form (ICF): formal agreement to participate in the study.
• Center characteristics: including name, location, number of beds, average annual HSCT volume, and types of procedures performed (adult, pediatric, autologous, allogeneic);
• ENT practices: including formula selection, caloric density, management of gastrointestinal symptoms, and the use of pediatric versus adult formulas.

**Figure 1:**
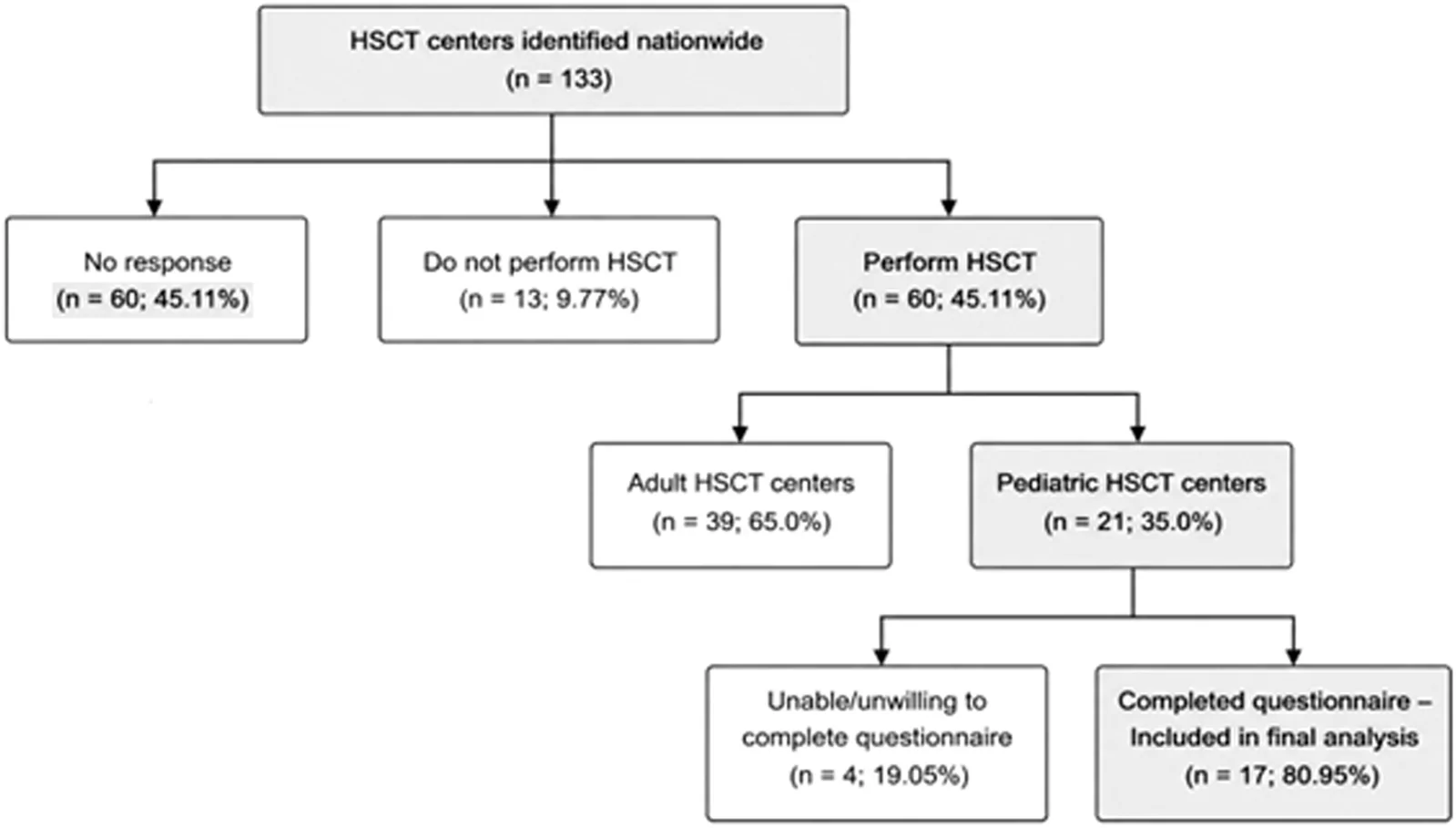
Flowchart illustrating the identification of hematopoietic stem cell transplantation centers, response rates, and questionnaire completion.

### Statistical analysis

All data were collected using Google Forms and exported to Microsoft Excel for analysis. Descriptive statistical analyses were performed using Excel tools, and categorical variables were summarized using absolute and relative frequencies.

The variables analyzed included characteristics of the transplant centers (region, number of beds, annual transplant volume, type of HSCT performed, whether the center conducts pediatric alone or both pediatric and adult HSCT), the professional completing the questionnaire, and practices related to ENT, including prophylactic nasoenteral tube placement, the timing of insertion, type and caloric density of enteral formulas, management of gastrointestinal symptoms affecting enteral nutrition tolerance, and the age at which adult formulas are introduced.

The main outcome was the characterization of ENT practices among pediatric HSCT centers in Brazil.

## Results

### Center identification

Among the 133 HSCT centers identified nationwide, 60 (45.11%) did not respond to the study invitation, while 13 (9.77%) reported not performing HSCT. Of the remaining 60 institutions performing HSCT, 21 (35.0%) reported performing pediatric HSCT and were considered eligible for the study, whereas 39 (65.0%) exclusively treated adult patients and were excluded. Among the eligible pediatric centers. Seventeen (81.0%) of the eligible centers completed the questionnaire, while four (19.0%) failed to do so (Table 1).

**Table 1:** Distribution of Hematopoietic Stem Cell Transplantation (HSCT) Centers Identified in Brazil, According to Region and Type of Transplantation.

	Does not perform HSCT	Performs Adult HSCT	Performs Pediatric HSCT	No response / Unable to contact	Total n (%)
**South**	-	10	6	4	20 (15.04)
RS	-	3	2	2	7 (5.26)
SC	-	2	-	-	2 (1.5)
PR	-	5	4	2	11 (8.27)
**Southeast**	9	23	12	41	85 (63.91)
SP	5	13	11	22	51 (38.35)
MG	2	3	-	11	16 (12.03)
RJ	2	7	1	8	18 (13.53)
**Midwest**	2	2	-	9	13 (9.77)
GOG GO	2	-	-	5	7 (5.26)
DF	-	2	-	4	6 (4.51)
**North**	1	-	-	1	2 (1.50)
PA	1	-	-	1	2 (1.50)
**Northeast**	1	4	3	5	13 (9.77)
RN	-	-	1	1	2 (1.5)
PB	-	1	-	-	1 (0.75)
PE	-	2	1	2	5 (3.76)
SE	1	-	-	-	1 (0.75)
BA	-	1	1	2	4 (3.01)
**Total** – n (%)	**13 (9.77)**	**39 (29.32)**	**21 (15.79)**	**60 (45.11)**	**133 (100)**

Bahia: BA; Federal District: DF; Goiás: GO; Mato Grosso do Sul: MS; Minas Gerais: MG; Pará: PA; Paraíba: PB; Paraná: PR; Pernambuco: PE; Rio de Janeiro: RJ; Rio Grande do Norte: RN; Rio Grande do Sul: RS; Santa Catarina: SC; São Paulo: SP; Sergipe: SE; Hematopoietic Stem Cell Transplantation: HSCT; Tocantins: TO

In terms of geographic distribution, most responses originated from the southeastern region, with seven hospitals and eight responses, all from the state of São Paulo. The higher number of responses is attributed to the participation of different teams within the same hospital. This was followed by the southern and northeastern regions. No responses were received from the northern or center-western regions, despite the identification of centers located in these areas (Table 1).

### Characteristics of transplant centers

The centers demonstrated regional variations in both the number of available beds and annual HSCT procedures. Most beds were located in the southeastern region (63.9%), followed by the South (15.0%) and the Northeast (9.8%). It is worth noting that some centers did not report the number of beds. Regarding the average annual number of transplants, three out of the 17 respondents were unable to provide this information. The Southeast accounted for the highest transplant volume (55%), followed by the Northeast (26.5%) and the South (18.4%). With respect to transplant type, 94.1% of centers performed both autologous and allogeneic HSCT, while one center conducted exclusively autologous procedures (Table 2).

**Table 2:** Characteristics of the pediatric Hematopoietic Stem Cell Transplantation (HSCT) centers that provided institutional data and professional profile of the respondents.

Region / state	Hospitals n	Beds n (%)	Number of transplants per year n (%)	Performs Pediatric and Adult HSCT n	Performs Pediatric HSCT only n	Performs Autologous and Allogeneic HSCT n	Performs Autologous HSCT only n	Professional	
								Nutritionist	Other
**Southern**	6	52 (32.5)	125 (18.41)	4	2	6	-	5	1**
RS	2	3*	6*	2		2	-	2	-
PR	4	49	49*	2	2	4	-	3	1
**Southeastern**	7	99 (61.9)	374 (55.08)	4	3	7	-	6	1**
SP	7	99	374*	4	3	7	-	6	1
**Northeastern**	2	9 (5.6)	180 (26.51)	1	1	1	1	2	-
RN	1	7	160	1	-	1	-	1	-
BA	1	2	20	-	1	-	1	1	-
**Total**	15	160	679	9	6	14	1	15 (88.24%)	2 (11.76%)

Bahia (BA); Paraná (PR); Rio Grande do Norte (RN); Rio Grande do Sul (RS); São Paulo (SP); Hematopoietic Stem Cell Transplantation (HSCT)

One of the hospitals in each of these states reported not knowing or not having this information.

** Physician and nurse.

The majority of the respondents (88.2%) were registered dietitians, while other participants included one nurse and one physician.

### Enteral nutritional therapy practices

An analysis of ENT practices revealed that 82.4% of centers routinely performed prophylactic nasoenteral tube placement in pediatric HSCT patients (Table 3). Most centers (52.94%) performed tube insertion by Day 0, while 29.41% reported doing so between Day 0 and Day +5. A minority of centers inserted the nasoenteral tube beyond this period. Pediatric polymeric formulas constituted the first-line choice in 82.35% of cases, with subsequent modifications to hydrolyzed or oligomeric formulas implemented as clinically indicated. None of the centers reported initiating ENT using amino acid-based formulas. Regarding caloric density, 82.4% of centers used normocaloric formulas as the first option, whereas 17.6% opted for hypercaloric formulas. Caloric density classification followed the criteria established by the Brazilian Society of Parenteral and Enteral Nutrition (BRASPEN) [9].

**Table 3:** Characteristics of nutritional therapy in Hematopoietic Stem Cell Transplantation (HSCT) centers.

**Is the nasoenteral tube inserted prophylactically?**	
Yes	14 (82.35%)
No	3 (17.65%)
**When used, at what time is the nasoenteral tube usually inserted?**	
Up to D0	9 (52.94%)
D0 to D+5 – 5	5 (29.41%)
D+5 to D+10	2 (11.76%)
D10 to D+15	0
D+16 – 1	1 (5.88%)
**What is the first-choice enteral formula at the start of enteral nutrition therapy?**	
Pediatric polymeric	14 (82.35%)
Oligomeric	2 (11.76%)
Extensively hydrolyzed	1 (5.88%)
Amino acids	0
**What is the first-choice formula concentration at the start of enteral nutrition therapy?**	
<0.9	0
0,9 - 1.2 kcal/ml	14 (82.35%)
>1.2 kcal/ml	3 (17.65%)
**In the presence of gastrointestinal symptoms affecting enteral diet tolerance (nausea, vomiting, mucositis, diarrhea), what is the most commonly used approach? (more than one option could be selected)**	
Reduce enteral diet volume	15 (30.61%)
Reduce caloric density	9 (18.37%)
Switch to hydrolyzed formula	13 (26.53%)
Combine enteral with parenteral nutrition	6 (12.24%)
Suspend enteral diet until symptoms subside	4 (8.16%)
Replace enteral nutrition with parenteral	2 (4.08%)
**Considering that in Brazil pediatric enteral formulas are recommended up to 10 years of age and there are no formulas specifically for adolescents, at what age does your center typically begin using adult formulas?**	
≥10 years	8 (47.06%)
>12 years	1 (5.88%)
>14 years	2 (11.76%)
>16 years	0
>18 years	1 (5.88%)
Do not use	4 (23.53%)
Others (response annulled)	1 (5.88)

**Source**: Viau, V.; Silva, A. *Questionnaire: Hematopoietic Stem Cell Transplantation Centers*. Developed by the authors.

In the presence of gastrointestinal symptoms compromising enteral diet tolerance, the most commonly reported strategies were volume reduction (30.6%), switching to hydrolyzed formulas (26.5%), and combining with parenteral nutrition (26.5%). This question allowed multiple responses; additional strategies are detailed in Table 3.

When considering formula selection for children aged ten years and older, practices varied across centers. Most (47.06%) reported using adult formulas from this age onward, while 23.53% did not use adult formulas for any patient under 18 years old. One response was excluded due to inconsistency with the question.

## Discussion

### Training of professionals responsible for enteral nutrition therapy in hematopoietic stem cell transplantation

According to the Collegiate Board Resolution (RDC) No. 503 (2021), hospitals performing ENT must have a multiprofessional nutrition therapy team (EMTN) composed of a physician, nutritionist, nurse, and pharmacist, all with specific training [10]. This study underscored the importance of multidisciplinary alignment to ensure adequate nutritional support. Although two respondents were non-nutrition specialists, their knowledge of EN practices suggests a degree of interdisciplinary familiarity with nutritional care protocols. However, this does not imply the absence of a registered dietitian in those centers, nor does it indicate that these professionals are the primary prescribers of nutritional therapy.

### Timing of Tube Insertion and Start of Enteral Nutrition Therapy

Early insertion of the nasoenteral tube and initiation of ENT are important components of nutritional support during pediatric HSCT, although these practices are still scarcely addressed in the literature. Typically, the first-line strategy involves the use of oral nutritional supplements; however, in HSCT patients, the anticipated decline in oral intake due to mucositis, gastrointestinal toxicity, and other adverse effects often justifies a more proactive approach. In this context, early nasoenteral tube placement aims to ensure timely nutritional support and minimize the risks associated with undernutrition during treatment [2,11,12].

This study revealed that 82.4% of centers perform prophylactic tube insertion by Day 0. Studies indicate that early ENT may improve feeding tolerance before the onset of mucositis, considering factors such as malnutrition and gastrointestinal toxicity. Beyond caloric support, early enteral nutrition (even when only trophic) may contribute to maintaining gut microbiota diversity and the integrity of the intestinal barrier, which are important in reducing infectious complications and systemic inflammation, particularly in immunocompromised patients such as those undergoing HSCT [13].

Following transplantation, adverse effects often hinder the implementation of ENT, making parenteral nutrition the only feasible option in some cases [2]. Although patients may initially meet their energy requirements orally, studies report a significant decline in intake during treatment, underscoring the importance of supplemental nutritional support to ensure adequate caloric intake [11,12]. Early initiation of enteral nutrition has also been associated with a reduction in gastrointestinal complications, highlighting its efficacy and safety compared to parenteral nutrition [14,15].

### First choice of enteral formula at the start of enteral nutrition therapy

This study showed that 82.4% of HSCT teams prioritize the use of pediatric polymeric enteral formulas, adjusting to hydrolyzed formulas according to gastric tolerance. These findings align with Langdana et al. [16], who emphasized the use of polymeric or semi-elemental formulas during conditioning or the first week of HSCT, reserving elemental formulas for cases of high toxicity.

Conversely, another study reported that 67.7% of patients required semi-elemental diets due to gastrointestinal toxicities but did not observe significant symptom improvement [17]. Although there are no specific guidelines for enteral formula choice in pediatric HSCT, the European Society of Paediatric and Neonatal Intensive Care recommends polymeric formulas as the first choice for critically ill children, reserving peptide-based formulas for cases of intolerance [18].

Most centers (82.35%) preferred normocaloric formulas (0.9-1.2 kcal/mL), which is consistent with European Society for Paediatric Gastroenterology, Hepatology and Nutrition (ESPGHAN) guidelines recommending formulas around 1 kcal/mL for most children, meeting both nutritional and hydration needs [19]. However, the ideal formula choice in pediatric HSCT remains challenging, since hypercaloric formulas may be indicated for increased nutritional needs but may not provide adequate fluid volumes.

### Hematopoietic stem cell transplantation side effects and management of enteral nutrition therapy

ENT is the preferred feeding route for children with cancer; however, HSCT-related side effects such as nausea, vomiting, mucositis, and diarrhea require careful monitoring. Enteral nutrition may be contraindicated in cases of hemodynamic instability or severe gastrointestinal complications [2,4]. Discontinuation of ENT may also be influenced by factors beyond feeding intolerance, which should be considered before interrupting therapy [4].

This study found that common strategies to manage gastrointestinal symptoms include reducing enteral nutrition volume, switching to hydrolyzed formulas, and lowering caloric density. As specific guidelines for these adjustments are limited, individualized evaluation is recommended to ensure nutritional adequacy and tolerance [7,20].

ENT is preferable to parenteral nutrition when the gastrointestinal tract is functional, as it reduces infectious and metabolic complications and helps maintain intestinal function [18]. However, clinical instability or gastrointestinal complications may limit ENT administration. In such situations, providing the largest tolerated amount of enteral nutrition, even if minimal, is recommended, and partial parenteral nutrition may be necessary to cover nutritional deficits [20].

The combined use of enteral nutrition and parenteral nutrition was reported by 26.53% of the teams in this study. Similar findings were observed in a study involving 65 pediatric patients undergoing allogeneic HSCT, where the combined use of enteral nutrition and parenteral nutrition was associated with a higher frequency of diarrhea, gastrointestinal complications, and graft-versus-host disease (GvHD). Parenteral nutrition was mainly indicated for patients with severe intestinal intolerance and diarrhea leading to discontinuation of enteral nutrition [14].

Guidelines recommend the use of parenteral nutrition when there is partial or total inability to use the gastrointestinal tract or when nutritional needs cannot be met enterally within a short period. Parenteral nutrition is essential in critical situations such as intense nausea, severe diarrhea, or mucositis [2,5,15] European Society for Clinical Nutrition and Metabolism (ESPEN) guidelines [21] recommend restricting parenteral nutrition to patients intolerant to ENT, avoiding routine use. Regarding diet suspension until symptom resolution, although criteria are unclear and studies are lacking, this practice may negatively impact the nutritional status of already at-risk patients [8,22].

Nutritional therapy monitoring is essential to assess tolerance and prevent complications, especially in severely ill children. Strategies, such as administration adjustments, diet formulation, and use of prokinetic agents, may help, although there is no clear evidence of their efficacy in HSCT. Multidisciplinary decisions should consider causes of intolerance before changes to avoid negative impacts on nutritional status [18,23,24].

Finally, the transition to adult enteral formulas in children over ten years old represents an additional challenge, especially due to the absence of specific products for this age group in Brazil. Although the Brazilian Society of Pediatrics recommends adult formulas from approximately 8 to 10 years onward [23], careful adjustment is necessary to ensure that pediatric nutritional requirements are met. Evidence also suggests that even when macronutrient needs are achieved through enteral or parenteral formulas, deficiencies in vitamins, minerals, and trace elements may still occur, reinforcing the importance of careful nutritional monitoring [24,25].

This study provides an overview of ENT practices among pediatric HSCT centers in Brazil and contributes to the limited literature investigating nutritional management in this population. By assessing centers across different regions of the country and describing their clinical practices, the study underscores important aspects of nutritional care during HSCT.

However, some limitations of this study should be considered. The data were obtained through a self-reported questionnaire, and the number of participating centers was limited compared to the total number of HSCT centers in Brazil. Despite these limitations, the study provides relevant insights into current practices and may contribute to the development of more standardized nutritional strategies in pediatric HSCT.

## Conclusion

This study found that ENT is the main strategy for nutritional support in children undergoing HSCT, with most centers adopting prophylactic nasoenteral tube insertion to help prevent nutritional deterioration during treatment. However, there is no consensus regarding the ideal enteral formula, and clinical decisions are often based on team experience and patient tolerance. Additionally, the limited availability of enteral formulas specifically designed for children aged 10–18 years represents an important challenge. These findings highlight the need for further studies and the development of guidelines to support standardized and evidence-based nutritional management in pediatric HSCT, aiming to ensure adequate nutritional support and improve clinical outcomes.

## Declaration of generative AI and AI-assisted technologies in the writing process

During the preparation of this work, the author(s) used ChatGPT (OpenAI) in order to assist with language editing and translation. After using this tool, the author(s) reviewed and edited the content as needed and take(s) full responsibility for the content of the publication.

## Data availability

The data that support the findings of this study are Available at the corresponding author upon reasonable request.

## Conflicts of interest

The authors declare that they have no conflicts of interest relevant to the manuscript.
